# Crystal structure and Hirshfeld surface analysis of a copper(II) complex with ethyl­enedi­amine and non-coordinated benzoate

**DOI:** 10.1107/S2056989019016669

**Published:** 2020-01-01

**Authors:** Adnan M. Qadir, Sevgi Kansiz, Georgina M. Rosair, Necmi Dege, Turganbay S. Iskenderov

**Affiliations:** a Department of Chemistry, College of Science, Salahaddin University, Erbil, Iraq; b Department of Physics, Faculty of Arts and Sciences, Ondokuz Mayıs University, 55139, Kurupelit, Samsun, Turkey; c Institute of Chemical Sciences, School of Engineering & Physical Sciences, Heriot-Watt University, Edinburgh, EH14 4AS, UK; d Department of Chemistry, Taras Shevchenko National University of Kyiv, 64, Vladimirska Str., Kiev 01601, Ukraine

**Keywords:** crystal structure, copper(II), ethyl­enedi­amine, 2-nitro­benzoate, Hirshfeld surface

## Abstract

In the title compound, di­aqua­bis­(ethyl­enedi­amine-κ^2^ *N*,*N*′)copper(II) bis­(2-nitro­benzoate), [Cu(C_2_H_8_N_2_)_2_(H_2_O)_2_](C_7_H_4_NO_4_)_2_, two di­aqua­bis­(ethyl­enedi­amine)­copper(II) cations and four nitro­benzoate anions are present in the asymmetric unit. All four anions are ‘whole-mol­ecule’ disordered over two sets of sites. The major components have refined occupancies of 0.572 (13), 0.591 (9), 0.601 (9) and 0.794 (10). The Cu^II^ ions exhibit slightly distorted octa­hedral geometries. In the crystal, cations and anions are connected to each other *via* N—H⋯O and O—H⋯O hydrogen bonds, forming a two-dimensional network parallel to (200). The inter­molecular contacts in the crystal were further analysed using Hirshfeld surface analysis, which indicates that the most significant contacts are O⋯H/H⋯O (42.9%), followed by H⋯H (35.7%), C⋯H/H⋯C (14.2%), C⋯C (2.9%), C⋯O/O⋯C (2.2%), N⋯H/H⋯N (0.9%) and N⋯O/O⋯N (0.3%).

## Abstract 2

The asymmetric unit of the title compound contains two di­aqua­bis­(ethyl­enedi­amine)­copper(II) cations and four nitro­benzoate anions. These are connected into three-mol­ecule aggregates *via* N—H⋯O and O—H⋯O hydrogen bonds. The anions are disordered and modelled in two orientations. The major conformations have occupancies of 57, 59, 60 and 79%

## Chemical context   

Carboxyl­ates are employed in the synthesis of new transition-metal complexes because they can stabilize them and additionally display different coordination modes such as monodendate, bidendate, bridging (*syn*–*syn*, *syn*–*anti* or *anti*–*anti* mode) and ionic. Copper(II) carboxyl­ates have been used as single precursors for the preparation of copper(II) oxide nanoparticles (Karthik & Qadir, 2019 ▸). Copper(II) complexes containing ethyl­enedi­amine derivatives and carboxyl­ate have shown anti­bacterial activity against pathogenic bacteria (Kumar *et al.*, 2013 ▸). It has been reported that some copper(II) carboxyl­ate complexes involving nitro­gen donor ligands exhibit carbonic anhydrase inhibitory activity (Dilek *et al.*, 2017 ▸). Ethyl­enedi­amine has good coordination and chelating ability, forming five-membered ring compounds with metal centers. Generally, these metallacycles display a twist conformation. Copper can take part in different biological processes. Thus, copper shows an important role in electron transfer, oxidation, and di­oxy­gen transport (Mirica *et al.*, 2004 ▸; Rosenzweig *et al.*, 2006 ▸). In this paper, we report the synthesis, single crystal structure determination and Hirshfeld surface analysis of a copper(II) complex containing ethyl­enedi­amine and 2-nitro­benzoate.
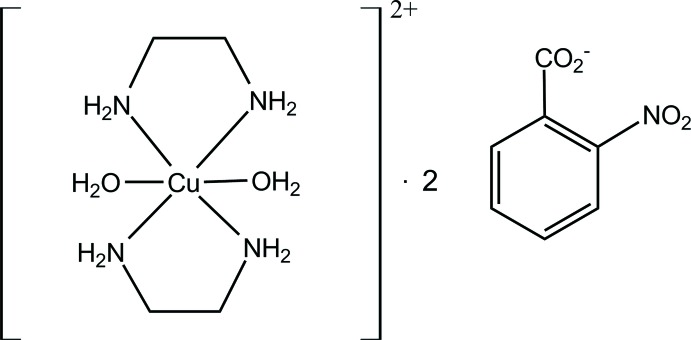


## Structural commentary   

**Figure 1:**
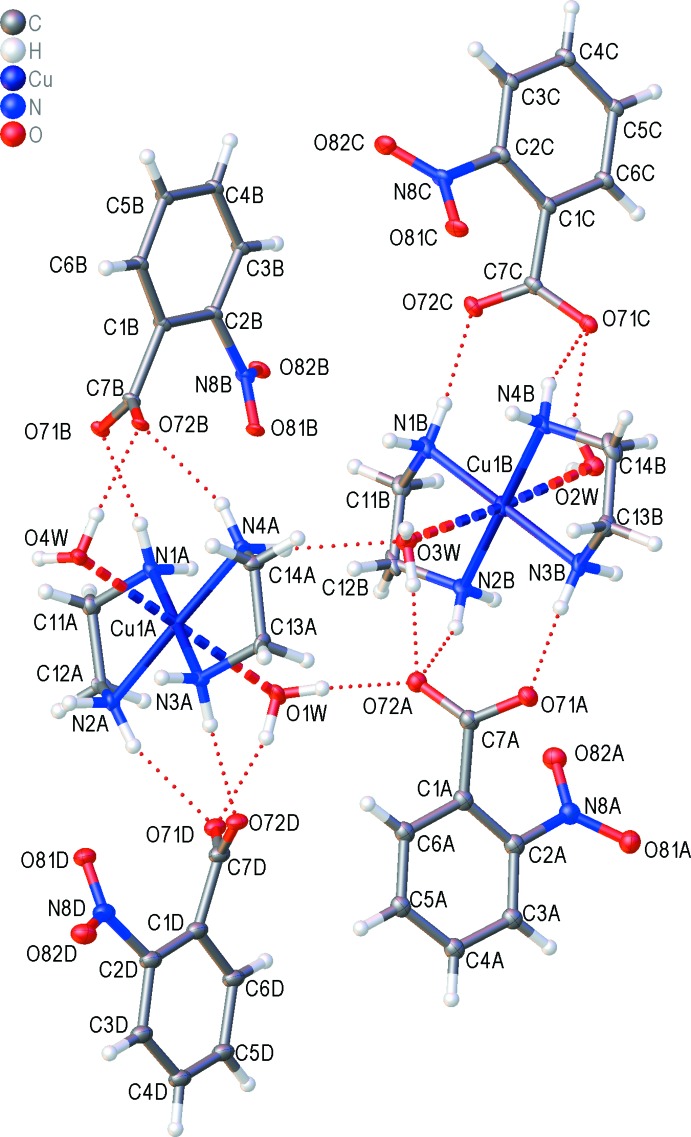
The asymmetric unit of the title complex, showing the major component of the disorder only, with the atom labeling. Displacement ellipsoids are drawn at the 50% probability level.

The asymmetric unit of title compound is shown in Fig. 1 ▸ and selected geometric parameters are given in Table 1 ▸. There are two independent bis­(ethyl­enedi­amine-*κ* ^2^ *N*)di­aqua­copper(II) cations and four 2-nitro­benzoate anions. In both cations, the Cu^II^ ion is coordinated by four N atoms of the ethyl­enedi­amine ligands which chelate the metal in the equatorial plane, and two axially coordinated water mol­ecules forming a slightly distorted octa­hedral geometry. The Cu—N bond lengths range from 1.991 (6) and 2.050 (5) Å (Table 1 ▸) and are similar to those observed in the structures containing [Cu(en)_2_(H_2_O)_2_]^2+^ cations (Kovbasyuk *et al.*, 1997 ▸; Gumienna-Kontecka *et al.*, 2007 ▸; Şen *et al.*, 2017 ▸). The axial Cu—O contacts in both conformers [2.599 (5) and 2.621 (5) Å for Cu1*A* and 2.557 (5) and 2.564 (5) Å for Cu1*B*] are noticeably longer than the equatorial Cu—N distances (Table 1 ▸) as a consequence of the Jahn–Teller effect (Kovbasyuk *et al.*, 1997 ▸). It is notable that although the complex cations are crystallographically non-centrosymmetric, the Cu—O bond lengths are very similar. In addition, the distances involving Cu1*A* and O are somewhat longer than for Cu1*B*. The N—O bond lengths [ranging from 1.223 (11) to 1.251 (15) Å] in the nitro group are close to the values observed for related compounds reported in the literature (Boulhaoua *et al.*, 2019 ▸; Kansız *et al.*, 2018 ▸, 2019 ▸).

## Supra­molecular features   

**Figure 2:**
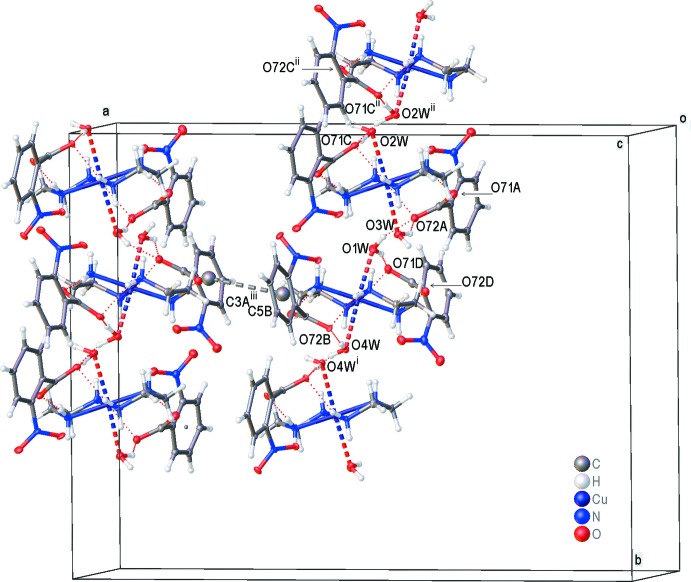
A view of the crystal packing of the title complex with only the major component of disorder shown and fine red dotted lines indicating hydrogen bonds (Table 2 ▸). Displacement ellipsoids are drawn at the 50% probability level [symmetry codes: (i) *x*, 1 − *y*, −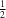 + *z*; (ii) *x*, −*y*, 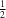 + *z*; (iii) 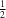 + *x*, 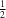 − *y*, 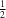 + *z*].

The crystal structure displays an extensive hydrogen-bonding network (Table 2 ▸). The crystal packing of the title compound (Fig. 2 ▸) features N—H⋯O and O—H⋯O hydrogen bonds, which connect the cations and anions, forming layers parallel to (200). All four water ligands are involved in inter­molecular hydrogen bonds. In addition, there are π–π stacking inter­actions with a centroid–centroid distance of 3.812 (6) Å between rings (C1*B*–C6*B*) and (C1*A*–C6*A*) at (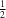 + *x*, 1/2 – *y*, 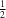 + *z*). These inter­actions consolidate the three-dimensional structure (Fig. 2 ▸).

## Hirshfeld surface analysis   

**Figure 3:**
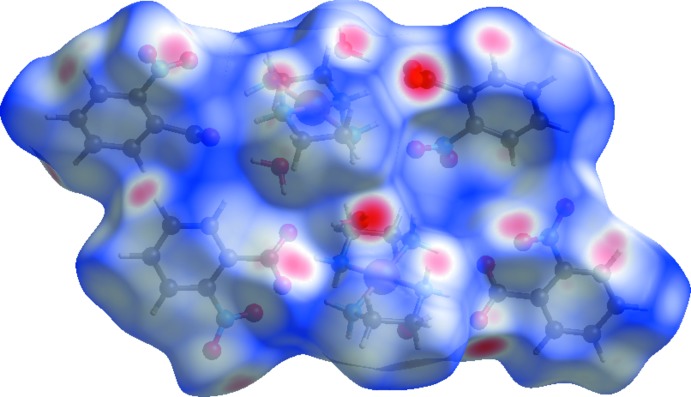
View of the Hirshfeld surface mapped over *d_norm_* in the range −0.6381 to +1.2243 (arbitrary units).

**Figure 4:**
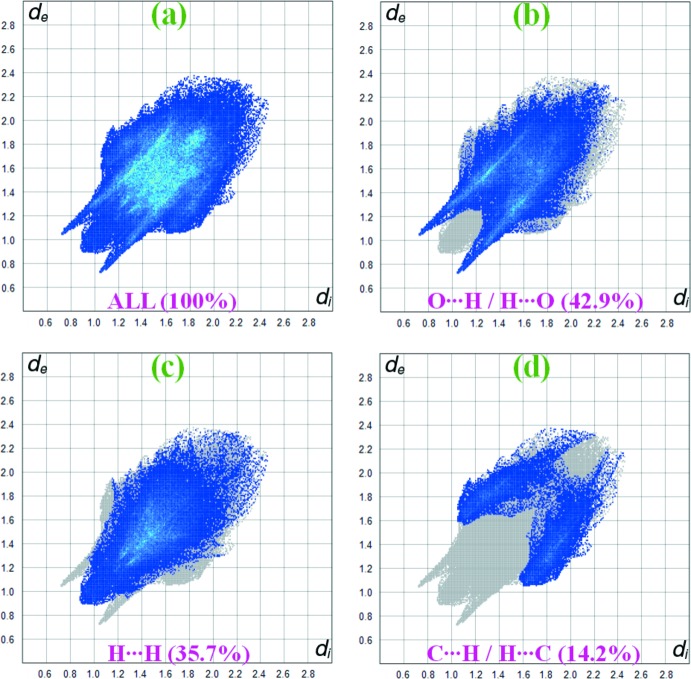
Hirshfeld surface fingerprint plots for the O⋯H/H⋯O, H⋯H and C⋯H/H⋯C contacts of the title complex.

In order to visualize the inter­molecular inter­actions in the crystal of the title compound, Hirshfeld surface analysis (Hirshfeld, 1977 ▸) was carried out by using *CrystalExplorer17.5* (Turner *et al.*, 2017 ▸). The Hirshfeld surface of the title complex plotted over *d_norm_* is shown in Fig. 3 ▸ where the N—H⋯O and O—H⋯O hydrogen bonds are indicated by red spots. Selected two-dimensional fingerprint plots are shown in Fig. 4 ▸ for all contacts as well as individual O⋯H/H⋯O, H⋯H and C⋯H/H⋯C contacts, whose percentage contribution is also given. The small percentage contributions from the other different inter­atomic contacts to the Hirshfeld surface are as follows: C⋯C (2.9%), C⋯O/O⋯C (2.2%), N⋯H/H⋯N (0.9%) and N⋯O/O⋯N (0.3%).

## Database survey   

A search of the Cambridge Structural Database (CSD, version 5.40, update of February 2019; Groom *et al.*, 2016 ▸) for the title complex revealed two similar structures: di­aqua­bis­(ethane-1,2-di­amine)­copper(II) 5-chloro-2-nitro­benzoate dihydrate (JUMGOP; Saini *et al.*, 2015 ▸) and *trans*-di­aqua­(1,3-di­amino­propane)­copper(II) 2-nitro­benzoate (WIFRUY; Sundberg & Klinga, 1994 ▸). Both complexes have an octa­hedral coordination geometry at the metal center and the Cu—N and Cu—O bond lengths in these structure are comparable to those in the title compound.

## Synthesis and crystallization   

An aqueous solution of sodium 2-nitro­benzoate (20 mmol, 3.78 g) was added to an aqueous solution of CuSO_4_·5H_2_O (10 mmol, 2.5 g) under stirring. The precipitate was filtered, dried and dissolved in a hot methanol solution containing ethyl­enedi­amine (20 mmol, 1.2 g) under stirring. The mixture was filtered and single crystals were obtained after slow evaporation for one week.

## Refinement   

Crystal data, data collection and structure refinement details are summarized in Table 3 ▸. The C-bound H atoms were positioned geometrically and refined using a riding model, with C—H = 0.95 and 0.99 Å with *U*_iso_(H) = 1.2*U*_eq_(C) for all C-bound H atoms. The N-bound H atoms were located in a difference-Fourier map and refined with N—H = 0.91 Å, and with *U*_iso_(H) = 1.5*U*_eq_(N). The H atoms bonded to O atoms (O1*W*, O2*W*, O3*W* and O4*W*) were located in a difference map and treated as part of a rigid group with oxygen as the pivot atom. All four anions are whole-mol­ecule disordered over two sets of sites. The major components have refined occupancies of 0.572 (13), 0.591 (9), 0.601 (9) and 794 (10). The major and minor components of disorder for each anion were constrained using the SAME command in *SHELXL* (Sheldrick, 2015 ▸). The SIMU command was used to apply restraints to the displacement parameters of the atoms of the anions.

## Supplementary Material

Crystal structure: contains datablock(s) I. DOI: 10.1107/S2056989019016669/lh5937sup1.cif

Structure factors: contains datablock(s) I. DOI: 10.1107/S2056989019016669/lh5937Isup2.hkl

CCDC reference: 1909170

Additional supporting information:  crystallographic information; 3D view; checkCIF report

## Figures and Tables

**Table 1:** Selected geometric parameters (Å, °)

N1*A*—Cu1*A*	1.991 (6)	N1*B*—Cu1*B*	1.999 (6)
Cu1*A*—N2*A*	2.044 (5)	Cu1*B*—N2*B*	2.050 (5)
Cu1*A*—N3*A*	2.012 (6)	Cu1*B*—N3*B*	2.004 (6)
Cu1*A*—N4*A*	2.017 (5)	Cu1*B*—N4*B*	2.031 (5)
Cu1*A*—O1*W*	2.621 (5)	Cu1*B*—O2*W*	2.557 (5)
Cu1*A*—O4*W*	2.599 (5)	Cu1*B*—O3*W*	2.564 (5)
			
N1*A*—Cu1*A*—N2*A*	85.1 (2)	N1*B*—Cu1*B*—N2*B*	84.8 (2)
N3*A*—Cu1*A*—N4*A*	85.2 (2)	N3*B*—Cu1*B*—N4*B*	85.0 (2)
O4*W*—Cu1*A*—O1*W*	177.6 (2)	O2*W*—Cu1*B*—O3*W*	176.3 (2)

**Table 2:** Hydrogen-bond geometry (Å, °)

*D*—H⋯*A*	*D*—H	H⋯*A*	*D*⋯*A*	*D*—H⋯*A*
N1*A*—H1*AA*⋯O71*B*	0.91	1.98	2.881 (14)	172
N3*A*—H3*AB*⋯O72*D*	0.91	1.90	2.809 (11)	174
N1*B*—H1*BB*⋯O72*C*	0.91	1.99	2.902 (8)	174
N2*B*—H2*BB*⋯O72*A*	0.91	2.02	2.874 (12)	155
N3*B*—H3*BA*⋯O71*A*	0.91	1.89	2.795 (10)	176
O1*W*—H1*WA*⋯O72*A*	0.85	1.97	2.794 (13)	164
O1*W*—H1*WB*⋯O71*D*	0.85	1.94	2.763 (19)	162
O2*W*—H2*WA*⋯O71*C*	0.87	1.98	2.739 (9)	145
O2*W*—H2*WB*⋯O71*C* ^i^	1.07	1.78	2.803 (10)	160
O3*W*—H3*WA*⋯O71*D* ^ii^	0.85	1.92	2.739 (15)	160
O3*W*—H3*WB*⋯O72*A*	0.85	2.04	2.750 (13)	141
O4*W*—H4*WA*⋯O72*B*	0.85	1.91	2.753 (12)	173
O4*W*—H4*WB*⋯O72*B* ^iii^	0.85	1.90	2.726 (13)	164

Symmetry codes: (i) 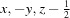; (ii) 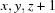; (iii) 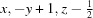.

**Table 3:** Experimental details

Crystal data	
Chemical formula	[Cu(C_2_H_8_N_2_)_2_(H_2_O)_2_](C_7_H_4_NO_4_)_2_
*M*_r_	552.00
Crystal system, space group	Monoclinic, *C* *c*
Temperature (K)	100
*a*, *b*, *c* (Å)	26.7742 (16), 20.8916 (14), 8.4254 (5)
β (°)	93.460 (3)
*V* (Å^3^)	4704.2 (5)
*Z*	8
Radiation type	Mo *K*α
μ (mm^−1^)	0.99
Crystal size (mm)	0.52 × 0.32 × 0.3

Data collection	
Diffractometer	Bruker APEXII CCD
Absorption correction	Multi-scan (*SADABS*; Bruker, 2013 ▸)
*T*_min_, *T*_max_	0.593, 0.746
No. of measured, independent and observed [*I* > 2σ(*I*)] reflections	44253, 13693, 11671
*R*_int_	0.038
(sin θ/λ)_max_ (Å^−1^)	0.746

Refinement	
*R*[*F* ^2^ > 2σ(*F* ^2^)], *wR*(*F* ^2^), *S*	0.056, 0.138, 1.07
No. of reflections	13693
No. of parameters	1026
No. of restraints	1750
H-atom treatment	H-atom parameters constrained
Δρ_max_, Δρ_min_ (e Å^−3^)	0.95, −0.86
Absolute structure	Refined as an inversion twin
Absolute structure parameter	0.49 (2)

Computer programs: *APEX2*and *SAINT* (Bruker, 2013 ▸), *SHELXS97* (Sheldrick, 2008 ▸), *SHELXL2018* (Sheldrick, 2015 ▸) and *OLEX2* (Dolomanov *et al.*, 2009 ▸).

## supplementary crystallographic information

### Crystal data

**Table:**

[Cu(C_2_H_8_N_2_)_2_(H_2_O)_2_](C_7_H_4_NO_4_)_2_	*F*(000) = 2296
*M**_r_* = 552.00	*D*_x_ = 1.559 Mg m^−^^3^
Monoclinic, *C**c*	Mo *K*α radiation, λ = 0.71073 Å
*a* = 26.7742 (16) Å	Cell parameters from 9966 reflections
*b* = 20.8916 (14) Å	θ = 3.0–32.0°
*c* = 8.4254 (5) Å	µ = 0.99 mm^−^^1^
β = 93.460 (3)°	*T* = 100 K
*V* = 4704.2 (5) Å^3^	Block, violet
*Z* = 8	0.52 × 0.32 × 0.3 mm

### Data collection

**Table:**

Bruker APEXII CCD diffractometer	13693 independent reflections
Radiation source: sealed tube	11671 reflections with *I* > 2σ(*I*)
Detector resolution: 8 pixels mm^-1^	*R*_int_ = 0.038
φ and ω scans	θ_max_ = 32.0°, θ_min_ = 1.2°
Absorption correction: multi-scan (SADABS; Bruker, 2013)	*h* = −36→39
*T*_min_ = 0.593, *T*_max_ = 0.746	*k* = −31→30
44253 measured reflections	*l* = −12→12

### Refinement

**Table:**

Refinement on *F*^2^	Hydrogen site location: mixed
Least-squares matrix: full	H-atom parameters constrained
*R*[*F*^2^ > 2σ(*F*^2^)] = 0.056	*w* = 1/[σ^2^(*F*_o_^2^) + (0.0259*P*)^2^ + 37.9856*P*] where *P* = (*F*_o_^2^ + 2*F*_c_^2^)/3
*wR*(*F*^2^) = 0.138	(Δ/σ)_max_ = 0.001
*S* = 1.07	Δρ_max_ = 0.95 e Å^−^^3^
13693 reflections	Δρ_min_ = −0.86 e Å^−^^3^
1026 parameters	Absolute structure: Refined as an inversion twin
1750 restraints	Absolute structure parameter: 0.49 (2)

### Special details

**Table:**

Geometry. All esds (except the esd in the dihedral angle between two l.s. planes) are estimated using the full covariance matrix. The cell esds are taken into account individually in the estimation of esds in distances, angles and torsion angles; correlations between esds in cell parameters are only used when they are defined by crystal symmetry. An approximate (isotropic) treatment of cell esds is used for estimating esds involving l.s. planes.
Refinement. Refined as a 2-component inversion twin.

### Fractional atomic coordinates and isotropic or equivalent isotropic displacement parameters (Å^2^)

**Table:**

	*x*	*y*	*z*	*U*_iso_*/*U*_eq_	Occ. (<1)
N1A	0.5784 (2)	0.3461 (2)	0.2462 (6)	0.0126 (10)	
H1AA	0.598599	0.365256	0.322980	0.015*	
H1AB	0.580381	0.302964	0.260497	0.015*	
Cu1A	0.50809 (3)	0.37528 (4)	0.26174 (6)	0.01011 (15)	
N2A	0.5082 (2)	0.3874 (3)	0.0210 (6)	0.0126 (10)	
H2AA	0.479364	0.371858	−0.027100	0.015*	
H2AB	0.510626	0.429707	−0.003059	0.015*	
N3A	0.4375 (2)	0.4064 (2)	0.2787 (6)	0.0117 (10)	
H3AA	0.436302	0.449744	0.268600	0.014*	
H3AB	0.417125	0.388898	0.199895	0.014*	
N4A	0.5077 (2)	0.3610 (3)	0.4985 (6)	0.0111 (9)	
H4AA	0.504922	0.318407	0.519411	0.013*	
H4AB	0.536709	0.375501	0.547333	0.013*	
C11A	0.5949 (3)	0.3633 (4)	0.0863 (9)	0.0162 (13)	
H11A	0.623899	0.336646	0.060565	0.019*	
H11B	0.605194	0.408813	0.084818	0.019*	
C12A	0.5518 (2)	0.3521 (3)	−0.0344 (7)	0.0118 (10)	
H12A	0.560315	0.367889	−0.140232	0.014*	
H12B	0.544170	0.305798	−0.042989	0.014*	
C13A	0.4207 (3)	0.3872 (4)	0.4358 (9)	0.0145 (12)	
H13A	0.409686	0.341955	0.432968	0.017*	
H13B	0.392168	0.414162	0.464280	0.017*	
C14A	0.4648 (2)	0.3958 (3)	0.5584 (7)	0.0147 (11)	
H14A	0.472947	0.441765	0.571734	0.018*	
H14B	0.456297	0.378310	0.662588	0.018*	
C1D	0.3508 (2)	0.3417 (3)	−0.2268 (7)	0.0171 (4)	0.572 (13)
C2D	0.3305 (5)	0.3975 (4)	−0.2818 (15)	0.0177 (5)	0.572 (13)
C3D	0.2951 (4)	0.4012 (6)	−0.4118 (13)	0.0180 (5)	0.572 (13)
H3D	0.281173	0.441178	−0.445140	0.022*	0.572 (13)
C4D	0.2814 (4)	0.3459 (7)	−0.4887 (12)	0.0179 (6)	0.572 (13)
H4D	0.256900	0.347113	−0.575055	0.021*	0.572 (13)
C5D	0.3017 (4)	0.2907 (6)	−0.4444 (12)	0.0177 (6)	0.572 (13)
H5D	0.292427	0.253116	−0.502416	0.021*	0.572 (13)
C6D	0.3366 (5)	0.2860 (5)	−0.3142 (13)	0.0175 (5)	0.572 (13)
H6D	0.350725	0.245659	−0.284658	0.021*	0.572 (13)
C7D	0.3826 (2)	0.3304 (3)	−0.0777 (7)	0.0165 (4)	0.572 (13)
N8D	0.3502 (4)	0.4560 (4)	−0.2127 (12)	0.0181 (6)	0.572 (13)
O71D	0.4229 (6)	0.2993 (13)	−0.096 (2)	0.0165 (9)	0.572 (13)
O72D	0.3693 (4)	0.3535 (7)	0.0484 (12)	0.0155 (9)	0.572 (13)
O81D	0.3947 (3)	0.4573 (5)	−0.1649 (12)	0.0191 (10)	0.572 (13)
O82D	0.3215 (3)	0.5017 (4)	−0.2149 (12)	0.0208 (10)	0.572 (13)
C1E	0.3508 (2)	0.3417 (3)	−0.2268 (7)	0.0171 (4)	0.428 (13)
C2E	0.3347 (7)	0.4043 (5)	−0.263 (2)	0.0177 (5)	0.428 (13)
C3E	0.3013 (5)	0.4194 (7)	−0.3942 (16)	0.0178 (6)	0.428 (13)
H3E	0.290727	0.462277	−0.412375	0.021*	0.428 (13)
C4E	0.2844 (5)	0.3710 (8)	−0.4958 (15)	0.0179 (6)	0.428 (13)
H4E	0.262588	0.380241	−0.586108	0.021*	0.428 (13)
C5E	0.2989 (5)	0.3119 (8)	−0.4652 (16)	0.0177 (6)	0.428 (13)
H5E	0.286754	0.278559	−0.533919	0.021*	0.428 (13)
C6E	0.3315 (6)	0.2964 (6)	−0.3355 (17)	0.0175 (5)	0.428 (13)
H6E	0.341044	0.252887	−0.320593	0.021*	0.428 (13)
C7E	0.3826 (2)	0.3304 (3)	−0.0777 (7)	0.0165 (4)	0.428 (13)
N8E	0.3549 (5)	0.4602 (6)	−0.1759 (17)	0.0182 (6)	0.428 (13)
O71E	0.4244 (8)	0.3014 (17)	−0.085 (3)	0.0165 (10)	0.428 (13)
O72E	0.3622 (5)	0.3489 (10)	0.0442 (16)	0.0156 (10)	0.428 (13)
O81E	0.3978 (4)	0.4578 (7)	−0.1178 (15)	0.0185 (10)	0.428 (13)
O82E	0.3282 (5)	0.5073 (5)	−0.1606 (16)	0.0203 (10)	0.428 (13)
N1B	0.5666 (2)	0.1555 (3)	0.7183 (6)	0.0148 (10)	
H1BA	0.567545	0.199005	0.720657	0.018*	
H1BB	0.587203	0.140328	0.799573	0.018*	
Cu1B	0.49668 (3)	0.12486 (4)	0.74192 (6)	0.01311 (18)	
N2B	0.4957 (2)	0.1094 (3)	0.5015 (6)	0.0140 (10)	
H2BA	0.496262	0.066684	0.480538	0.017*	
H2BB	0.467456	0.126368	0.452622	0.017*	
N3B	0.4264 (2)	0.0950 (3)	0.7658 (6)	0.0141 (10)	
H3BA	0.405717	0.111828	0.686888	0.017*	
H3BB	0.424948	0.051583	0.758559	0.017*	
N4B	0.4984 (2)	0.1396 (3)	0.9805 (6)	0.0136 (10)	
H4BA	0.527141	0.123326	1.027803	0.016*	
H4BB	0.497220	0.182217	1.001913	0.016*	
C11B	0.5830 (3)	0.1318 (3)	0.5646 (9)	0.0154 (13)	
H11C	0.591797	0.085852	0.573045	0.019*	
H11D	0.612888	0.155762	0.534459	0.019*	
C12B	0.5411 (3)	0.1412 (3)	0.4428 (7)	0.0171 (13)	
H12C	0.549669	0.122034	0.340388	0.021*	
H12D	0.534752	0.187439	0.425888	0.021*	
C13B	0.4103 (3)	0.1158 (4)	0.9221 (9)	0.0161 (13)	
H13C	0.381676	0.089673	0.953369	0.019*	
H13D	0.399889	0.161305	0.917818	0.019*	
C14B	0.4548 (3)	0.1071 (4)	1.0404 (8)	0.0221 (14)	
H14C	0.447085	0.125292	1.144595	0.027*	
H14D	0.462008	0.060904	1.055028	0.027*	
C1B	0.6625 (2)	0.4018 (2)	0.7429 (6)	0.0102 (4)	0.591 (19)
C2B	0.6758 (2)	0.3377 (2)	0.7682 (6)	0.0106 (4)	0.591 (19)
C3B	0.7066 (4)	0.3136 (4)	0.8906 (10)	0.0107 (5)	0.591 (19)
H3B	0.713114	0.269087	0.901114	0.013*	0.591 (19)
C4B	0.7276 (4)	0.3579 (5)	0.9978 (10)	0.0108 (5)	0.591 (19)
H4B	0.750458	0.344503	1.081797	0.013*	0.591 (19)
C5B	0.7145 (4)	0.4224 (5)	0.9804 (11)	0.0107 (5)	0.591 (19)
H5B	0.728121	0.452487	1.055735	0.013*	0.591 (19)
C6B	0.6825 (5)	0.4438 (4)	0.8574 (12)	0.0106 (5)	0.591 (19)
H6B	0.674071	0.487955	0.850870	0.013*	0.591 (19)
C7B	0.6310 (7)	0.4229 (13)	0.5960 (10)	0.0102 (5)	0.591 (19)
N8B	0.6529 (4)	0.2899 (4)	0.6573 (11)	0.0111 (5)	0.591 (19)
O71B	0.6474 (5)	0.4129 (6)	0.4634 (13)	0.0105 (9)	0.591 (19)
O72B	0.5899 (4)	0.4499 (6)	0.6186 (14)	0.0098 (9)	0.591 (19)
O81B	0.6092 (4)	0.2968 (6)	0.6063 (12)	0.0109 (9)	0.591 (19)
O82B	0.6781 (3)	0.2426 (4)	0.6213 (12)	0.0131 (9)	0.591 (19)
C1G	0.6625 (2)	0.4018 (2)	0.7429 (6)	0.0102 (4)	0.409 (19)
C2G	0.6758 (2)	0.3377 (2)	0.7682 (6)	0.0106 (4)	0.409 (19)
C3G	0.7112 (6)	0.3263 (7)	0.8914 (15)	0.0108 (5)	0.409 (19)
H3G	0.723105	0.283923	0.909890	0.013*	0.409 (19)
C4G	0.7297 (5)	0.3758 (7)	0.9888 (16)	0.0110 (5)	0.409 (19)
H4G	0.754418	0.366033	1.070851	0.013*	0.409 (19)
C5G	0.7137 (6)	0.4388 (7)	0.9714 (16)	0.0108 (5)	0.409 (19)
H5G	0.725074	0.471568	1.042973	0.013*	0.409 (19)
C6G	0.6801 (8)	0.4509 (5)	0.8428 (18)	0.0105 (5)	0.409 (19)
H6G	0.668933	0.493466	0.822825	0.013*	0.409 (19)
C7G	0.6322 (10)	0.4214 (18)	0.5917 (14)	0.0101 (5)	0.409 (19)
N8G	0.6527 (5)	0.2851 (5)	0.6764 (16)	0.0111 (5)	0.409 (19)
O71G	0.6525 (7)	0.4050 (8)	0.4693 (18)	0.0103 (10)	0.409 (19)
O72G	0.5939 (6)	0.4560 (8)	0.603 (2)	0.0097 (10)	0.409 (19)
O81G	0.6082 (5)	0.2910 (9)	0.6290 (18)	0.0112 (10)	0.409 (19)
O82G	0.6783 (4)	0.2361 (5)	0.6735 (18)	0.0128 (10)	0.409 (19)
C1A	0.3417 (2)	0.1623 (2)	0.2578 (6)	0.0152 (4)	0.601 (9)
C2A	0.3179 (5)	0.1062 (4)	0.2068 (14)	0.0158 (5)	0.601 (9)
C3A	0.2819 (4)	0.1031 (5)	0.0828 (11)	0.0157 (6)	0.601 (9)
H3A	0.265887	0.063868	0.053660	0.019*	0.601 (9)
C4A	0.2702 (4)	0.1593 (5)	0.0027 (11)	0.0159 (6)	0.601 (9)
H4A	0.245408	0.159014	−0.083011	0.019*	0.601 (9)
C5A	0.2941 (4)	0.2164 (5)	0.0459 (11)	0.0157 (6)	0.601 (9)
H5A	0.286164	0.254408	−0.012146	0.019*	0.601 (9)
C6A	0.3296 (5)	0.2183 (4)	0.1730 (12)	0.0154 (5)	0.601 (9)
H6A	0.345530	0.257556	0.202300	0.018*	0.601 (9)
C7A	0.3746 (5)	0.1699 (8)	0.4095 (10)	0.0153 (6)	0.601 (9)
N8A	0.3354 (3)	0.0455 (4)	0.2762 (11)	0.0171 (6)	0.601 (9)
O71A	0.3590 (4)	0.1453 (4)	0.5321 (10)	0.0160 (9)	0.601 (9)
O72A	0.4171 (4)	0.1964 (5)	0.3989 (16)	0.0155 (10)	0.601 (9)
O81A	0.3057 (3)	0.0005 (3)	0.2881 (11)	0.0202 (9)	0.601 (9)
O82A	0.3802 (3)	0.0417 (4)	0.3236 (10)	0.0193 (9)	0.601 (9)
C1F	0.3417 (2)	0.1623 (2)	0.2578 (6)	0.0152 (4)	0.399 (9)
C2F	0.3231 (7)	0.1000 (4)	0.225 (2)	0.0159 (5)	0.399 (9)
C3F	0.2893 (6)	0.0852 (7)	0.0989 (16)	0.0159 (6)	0.399 (9)
H3F	0.277381	0.042728	0.083386	0.019*	0.399 (9)
C4F	0.2734 (6)	0.1340 (7)	−0.0043 (16)	0.0159 (6)	0.399 (9)
H4F	0.250795	0.125302	−0.092963	0.019*	0.399 (9)
C5F	0.2910 (6)	0.1957 (7)	0.0244 (16)	0.0157 (6)	0.399 (9)
H5F	0.279348	0.229331	−0.044119	0.019*	0.399 (9)
C6F	0.3252 (7)	0.2097 (6)	0.1502 (17)	0.0154 (6)	0.399 (9)
H6F	0.337521	0.252187	0.163207	0.018*	0.399 (9)
C7F	0.3737 (7)	0.1752 (12)	0.4088 (13)	0.0153 (6)	0.399 (9)
N8F	0.3431 (4)	0.0451 (5)	0.3204 (16)	0.0169 (6)	0.399 (9)
O71F	0.3543 (5)	0.1626 (6)	0.5361 (15)	0.0152 (10)	0.399 (9)
O72F	0.4133 (6)	0.2073 (7)	0.393 (2)	0.0154 (10)	0.399 (9)
O81F	0.3166 (4)	−0.0008 (5)	0.3512 (16)	0.0190 (9)	0.399 (9)
O82F	0.3868 (4)	0.0483 (6)	0.3765 (15)	0.0187 (10)	0.399 (9)
C1C	0.6565 (2)	0.0820 (2)	1.2352 (7)	0.0121 (4)	0.794 (10)
C2C	0.6821 (3)	0.1344 (3)	1.3002 (9)	0.0125 (5)	0.794 (10)
C3C	0.7183 (3)	0.1298 (3)	1.4243 (8)	0.0126 (5)	0.794 (10)
H3C	0.734927	0.167092	1.464820	0.015*	0.794 (10)
C4C	0.7299 (3)	0.0706 (4)	1.4880 (8)	0.0126 (5)	0.794 (10)
H4C	0.755042	0.066097	1.571790	0.015*	0.794 (10)
C5C	0.7036 (3)	0.0167 (3)	1.4261 (8)	0.0124 (5)	0.794 (10)
H5C	0.710592	−0.024318	1.470813	0.015*	0.794 (10)
C6C	0.6679 (4)	0.0225 (3)	1.3015 (9)	0.0121 (5)	0.794 (10)
H6C	0.650997	−0.014499	1.260819	0.014*	0.794 (10)
C7C	0.6219 (4)	0.0840 (7)	1.0855 (12)	0.0123 (5)	0.794 (10)
N8C	0.6677 (3)	0.1993 (3)	1.2447 (8)	0.0133 (5)	0.794 (10)
O71C	0.5823 (3)	0.0503 (3)	1.0884 (10)	0.0129 (9)	0.794 (10)
O72C	0.6380 (3)	0.1118 (3)	0.9677 (8)	0.0142 (9)	0.794 (10)
O81C	0.6224 (2)	0.2084 (2)	1.2129 (7)	0.0151 (8)	0.794 (10)
O82C	0.7004 (2)	0.2407 (2)	1.2412 (7)	0.0172 (8)	0.794 (10)
C1H	0.6565 (2)	0.0820 (2)	1.2352 (7)	0.0121 (4)	0.206 (10)
C2H	0.6740 (11)	0.1416 (6)	1.285 (3)	0.0126 (5)	0.206 (10)
C3H	0.7100 (10)	0.1454 (11)	1.409 (3)	0.0126 (5)	0.206 (10)
H3H	0.725396	0.185376	1.433505	0.015*	0.206 (10)
C4H	0.7237 (10)	0.0926 (12)	1.497 (3)	0.0126 (6)	0.206 (10)
H4H	0.746643	0.096325	1.586691	0.015*	0.206 (10)
C5H	0.7034 (11)	0.0325 (11)	1.451 (3)	0.0124 (6)	0.206 (10)
H5H	0.713171	−0.004612	1.510462	0.015*	0.206 (10)
C6H	0.6692 (15)	0.0272 (8)	1.321 (4)	0.0122 (5)	0.206 (10)
H6H	0.654864	−0.013013	1.291386	0.015*	0.206 (10)
C7H	0.6201 (15)	0.082 (3)	1.088 (4)	0.0124 (5)	0.206 (10)
N8H	0.6538 (8)	0.2019 (8)	1.211 (3)	0.0135 (6)	0.206 (10)
O71H	0.5758 (9)	0.0596 (13)	1.097 (4)	0.0124 (10)	0.206 (10)
O72H	0.6288 (10)	0.1206 (12)	0.978 (3)	0.0128 (10)	0.206 (10)
O81H	0.6090 (8)	0.2047 (10)	1.160 (3)	0.0144 (10)	0.206 (10)
O82H	0.6831 (9)	0.2480 (9)	1.210 (3)	0.0151 (9)	0.206 (10)
O1W	0.47408 (19)	0.2612 (2)	0.1831 (6)	0.0168 (10)	
H1WA	0.461064	0.235558	0.247176	0.025*	
H1WB	0.452828	0.270177	0.107484	0.025*	
O2W	0.52864 (19)	0.0129 (2)	0.8172 (6)	0.0156 (9)	
H2WA	0.551709	0.034405	0.872122	0.023*	
H2WB	0.555603	−0.004257	0.739509	0.023*	
O3W	0.47048 (19)	0.2395 (2)	0.6668 (6)	0.0154 (9)	
H3WA	0.457633	0.266081	0.728823	0.023*	
H3WB	0.443693	0.236396	0.608247	0.023*	
O4W	0.53815 (18)	0.4895 (2)	0.3438 (5)	0.0133 (9)	
H4WA	0.553913	0.480466	0.431416	0.020*	
H4WB	0.558611	0.503253	0.278334	0.020*	

### Atomic displacement parameters (Å^2^)

**Table:**

	*U*^11^	*U*^22^	*U*^33^	*U*^12^	*U*^13^	*U*^23^
N1A	0.017 (3)	0.013 (2)	0.009 (2)	0.0022 (18)	0.0027 (17)	0.0001 (16)
Cu1A	0.0101 (4)	0.0129 (3)	0.0077 (3)	0.0029 (2)	0.0033 (3)	0.00127 (19)
N2A	0.012 (2)	0.014 (2)	0.012 (2)	0.000 (2)	0.0029 (18)	0.0035 (19)
N3A	0.013 (2)	0.013 (2)	0.009 (2)	0.0027 (17)	0.0003 (17)	−0.0020 (16)
N4A	0.010 (2)	0.012 (2)	0.012 (2)	0.0010 (19)	0.0023 (17)	0.0009 (18)
C11A	0.015 (3)	0.019 (3)	0.015 (3)	−0.001 (2)	0.003 (2)	0.003 (2)
C12A	0.008 (2)	0.019 (3)	0.008 (2)	0.004 (2)	0.0043 (18)	−0.001 (2)
C13A	0.011 (3)	0.017 (3)	0.016 (3)	−0.003 (2)	0.007 (2)	−0.002 (2)
C14A	0.012 (3)	0.022 (3)	0.010 (2)	−0.002 (2)	0.0011 (19)	−0.002 (2)
C1D	0.0134 (7)	0.0233 (8)	0.0143 (7)	−0.0031 (7)	−0.0007 (7)	−0.0010 (7)
C2D	0.0137 (9)	0.0239 (9)	0.0151 (9)	−0.0026 (9)	−0.0010 (9)	−0.0005 (9)
C3D	0.0137 (10)	0.0243 (11)	0.0157 (10)	−0.0028 (10)	−0.0012 (9)	−0.0008 (10)
C4D	0.0134 (11)	0.0242 (11)	0.0158 (11)	−0.0031 (11)	−0.0016 (10)	−0.0011 (11)
C5D	0.0134 (10)	0.0241 (11)	0.0154 (10)	−0.0030 (10)	−0.0016 (10)	−0.0010 (10)
C6D	0.0134 (10)	0.0239 (10)	0.0149 (10)	−0.0031 (9)	−0.0011 (9)	−0.0010 (9)
C7D	0.0137 (8)	0.0219 (8)	0.0135 (8)	−0.0031 (7)	−0.0007 (7)	−0.0014 (7)
N8D	0.0145 (11)	0.0238 (11)	0.0158 (12)	−0.0016 (10)	−0.0010 (11)	0.0003 (11)
O71D	0.0142 (16)	0.0215 (17)	0.0137 (17)	−0.0022 (15)	0.0000 (15)	−0.0023 (16)
O72D	0.0141 (18)	0.0201 (17)	0.0121 (15)	−0.0014 (17)	−0.0014 (15)	−0.0031 (14)
O81D	0.0166 (17)	0.0225 (17)	0.0177 (19)	−0.0033 (16)	−0.0034 (18)	0.0004 (18)
O82D	0.0174 (17)	0.0260 (17)	0.0187 (19)	0.0022 (16)	−0.0016 (17)	−0.0008 (18)
C1E	0.0134 (7)	0.0233 (8)	0.0143 (7)	−0.0031 (7)	−0.0007 (7)	−0.0010 (7)
C2E	0.0137 (9)	0.0239 (9)	0.0152 (9)	−0.0026 (9)	−0.0010 (9)	−0.0005 (9)
C3E	0.0135 (10)	0.0242 (11)	0.0156 (10)	−0.0027 (10)	−0.0013 (10)	−0.0008 (10)
C4E	0.0134 (11)	0.0244 (11)	0.0156 (11)	−0.0028 (11)	−0.0015 (10)	−0.0008 (11)
C5E	0.0134 (10)	0.0241 (11)	0.0153 (10)	−0.0031 (10)	−0.0014 (9)	−0.0009 (10)
C6E	0.0134 (10)	0.0239 (10)	0.0148 (10)	−0.0031 (10)	−0.0010 (9)	−0.0009 (10)
C7E	0.0137 (8)	0.0219 (8)	0.0135 (8)	−0.0031 (7)	−0.0007 (7)	−0.0014 (7)
N8E	0.0146 (11)	0.0238 (11)	0.0160 (12)	−0.0017 (10)	−0.0009 (11)	0.0000 (11)
O71E	0.0143 (17)	0.0215 (17)	0.0136 (18)	−0.0022 (16)	0.0001 (16)	−0.0021 (16)
O72E	0.0142 (18)	0.0201 (17)	0.0122 (16)	−0.0024 (17)	−0.0009 (16)	−0.0027 (15)
O81E	0.0157 (18)	0.0229 (17)	0.016 (2)	−0.0028 (17)	−0.0025 (19)	0.0005 (19)
O82E	0.0169 (18)	0.0256 (18)	0.0181 (19)	0.0017 (17)	−0.0011 (18)	−0.0008 (19)
N1B	0.022 (3)	0.012 (2)	0.012 (2)	0.0015 (19)	0.0041 (19)	0.0002 (16)
Cu1B	0.0176 (5)	0.0136 (3)	0.0087 (4)	−0.0001 (2)	0.0056 (3)	0.0000 (2)
N2B	0.018 (3)	0.016 (2)	0.008 (2)	0.001 (2)	0.0033 (18)	−0.0025 (19)
N3B	0.017 (3)	0.013 (2)	0.014 (2)	−0.0024 (18)	0.0077 (18)	−0.0003 (17)
N4B	0.021 (3)	0.011 (2)	0.009 (2)	0.003 (2)	0.0010 (18)	0.0019 (18)
C11B	0.018 (3)	0.016 (3)	0.013 (3)	0.000 (2)	0.007 (2)	−0.003 (2)
C12B	0.031 (4)	0.011 (3)	0.010 (2)	0.000 (2)	0.008 (2)	0.001 (2)
C13B	0.017 (3)	0.019 (3)	0.014 (3)	−0.003 (2)	0.008 (2)	−0.002 (2)
C14B	0.033 (4)	0.020 (3)	0.015 (3)	0.003 (3)	0.012 (3)	0.002 (2)
C1B	0.0092 (7)	0.0119 (7)	0.0095 (7)	0.0040 (6)	0.0003 (6)	0.0026 (6)
C2B	0.0096 (7)	0.0119 (7)	0.0101 (7)	0.0043 (6)	0.0003 (6)	0.0026 (6)
C3B	0.0097 (9)	0.0118 (10)	0.0106 (9)	0.0041 (9)	0.0001 (8)	0.0031 (9)
C4B	0.0099 (9)	0.0118 (11)	0.0106 (9)	0.0041 (10)	−0.0002 (9)	0.0035 (10)
C5B	0.0097 (9)	0.0119 (10)	0.0104 (9)	0.0043 (10)	−0.0001 (9)	0.0034 (10)
C6B	0.0095 (9)	0.0120 (10)	0.0101 (9)	0.0041 (9)	0.0004 (8)	0.0029 (9)
C7B	0.0092 (10)	0.0118 (10)	0.0094 (9)	0.0036 (9)	0.0002 (9)	0.0025 (9)
N8B	0.0108 (9)	0.0120 (10)	0.0105 (10)	0.0040 (9)	0.0006 (9)	0.0026 (9)
O71B	0.0099 (18)	0.0122 (18)	0.0094 (15)	0.0045 (15)	0.0008 (15)	0.0018 (15)
O72B	0.0082 (16)	0.0122 (17)	0.0086 (17)	0.0032 (15)	−0.0018 (15)	0.0031 (15)
O81B	0.0097 (15)	0.0121 (17)	0.0108 (18)	0.0013 (14)	−0.0018 (15)	0.0034 (16)
O82B	0.0140 (16)	0.0132 (16)	0.0122 (18)	0.0065 (14)	0.0010 (17)	0.0018 (16)
C1G	0.0092 (7)	0.0119 (7)	0.0095 (7)	0.0040 (6)	0.0003 (6)	0.0026 (6)
C2G	0.0096 (7)	0.0119 (7)	0.0101 (7)	0.0043 (6)	0.0003 (6)	0.0026 (6)
C3G	0.0099 (9)	0.0118 (10)	0.0107 (9)	0.0043 (9)	0.0001 (8)	0.0030 (9)
C4G	0.0100 (10)	0.0120 (11)	0.0108 (9)	0.0043 (10)	0.0000 (9)	0.0034 (10)
C5G	0.0098 (10)	0.0119 (11)	0.0105 (9)	0.0043 (10)	0.0000 (9)	0.0034 (10)
C6G	0.0095 (9)	0.0120 (10)	0.0101 (9)	0.0042 (9)	0.0003 (8)	0.0031 (9)
C7G	0.0092 (10)	0.0118 (10)	0.0094 (9)	0.0037 (9)	0.0002 (9)	0.0025 (9)
N8G	0.0107 (9)	0.0120 (10)	0.0106 (10)	0.0041 (9)	0.0004 (9)	0.0026 (9)
O71G	0.0098 (18)	0.0120 (19)	0.0091 (16)	0.0047 (17)	0.0008 (16)	0.0021 (16)
O72G	0.0078 (17)	0.0124 (18)	0.0086 (18)	0.0028 (16)	−0.0013 (16)	0.0028 (16)
O81G	0.0105 (16)	0.0123 (18)	0.0101 (19)	0.0023 (16)	−0.0030 (17)	0.0037 (17)
O82G	0.0135 (17)	0.0127 (17)	0.0120 (19)	0.0061 (16)	−0.0003 (18)	0.0025 (17)
C1A	0.0139 (8)	0.0167 (9)	0.0152 (8)	0.0021 (8)	0.0027 (7)	0.0013 (8)
C2A	0.0145 (10)	0.0171 (10)	0.0162 (10)	0.0021 (9)	0.0028 (9)	0.0010 (9)
C3A	0.0141 (11)	0.0174 (11)	0.0158 (10)	0.0023 (10)	0.0025 (10)	0.0011 (10)
C4A	0.0143 (11)	0.0177 (12)	0.0158 (11)	0.0025 (11)	0.0027 (10)	0.0014 (11)
C5A	0.0142 (11)	0.0174 (12)	0.0158 (11)	0.0024 (11)	0.0028 (10)	0.0016 (10)
C6A	0.0140 (10)	0.0170 (11)	0.0153 (10)	0.0023 (10)	0.0029 (9)	0.0015 (10)
C7A	0.0143 (11)	0.0167 (12)	0.0150 (10)	0.0018 (10)	0.0023 (9)	0.0014 (10)
N8A	0.0159 (11)	0.0176 (11)	0.0180 (12)	0.0019 (10)	0.0022 (10)	0.0006 (11)
O71A	0.0163 (17)	0.0181 (19)	0.0137 (15)	0.0012 (17)	0.0013 (15)	0.0026 (17)
O72A	0.0138 (17)	0.0167 (19)	0.0160 (16)	0.0003 (17)	0.0017 (15)	0.0011 (17)
O81A	0.0197 (18)	0.0190 (16)	0.0218 (18)	−0.0011 (16)	0.0009 (17)	0.0018 (17)
O82A	0.0178 (16)	0.0184 (15)	0.0215 (17)	0.0037 (15)	−0.0005 (16)	0.0005 (16)
C1F	0.0139 (8)	0.0167 (9)	0.0152 (8)	0.0021 (8)	0.0027 (7)	0.0013 (8)
C2F	0.0145 (10)	0.0172 (10)	0.0161 (10)	0.0020 (9)	0.0027 (9)	0.0009 (9)
C3F	0.0145 (11)	0.0175 (11)	0.0160 (11)	0.0019 (10)	0.0028 (10)	0.0011 (10)
C4F	0.0144 (11)	0.0175 (12)	0.0159 (11)	0.0020 (11)	0.0027 (10)	0.0013 (11)
C5F	0.0143 (11)	0.0174 (12)	0.0156 (11)	0.0022 (11)	0.0028 (10)	0.0014 (11)
C6F	0.0141 (10)	0.0170 (11)	0.0154 (10)	0.0021 (10)	0.0028 (9)	0.0014 (10)
C7F	0.0143 (11)	0.0167 (12)	0.0150 (10)	0.0019 (10)	0.0023 (9)	0.0013 (10)
N8F	0.0158 (12)	0.0174 (11)	0.0177 (12)	0.0018 (10)	0.0025 (11)	0.0006 (11)
O71F	0.0152 (18)	0.017 (2)	0.0137 (16)	0.0029 (18)	0.0028 (16)	0.0014 (18)
O72F	0.0139 (18)	0.016 (2)	0.0159 (17)	0.0012 (18)	0.0017 (16)	0.0013 (18)
O81F	0.0180 (17)	0.0185 (16)	0.0206 (18)	0.0004 (16)	0.0024 (16)	0.0011 (17)
O82F	0.0183 (18)	0.0184 (18)	0.0196 (19)	0.0045 (17)	0.0023 (18)	0.0002 (18)
C1C	0.0137 (8)	0.0106 (7)	0.0120 (7)	0.0051 (7)	0.0002 (7)	0.0010 (6)
C2C	0.0139 (10)	0.0106 (8)	0.0129 (9)	0.0055 (8)	−0.0003 (8)	0.0009 (8)
C3C	0.0138 (10)	0.0109 (10)	0.0128 (9)	0.0057 (9)	−0.0002 (9)	0.0004 (9)
C4C	0.0137 (11)	0.0112 (10)	0.0129 (10)	0.0055 (10)	−0.0001 (9)	0.0004 (9)
C5C	0.0134 (10)	0.0111 (10)	0.0126 (10)	0.0052 (10)	0.0001 (9)	0.0006 (9)
C6C	0.0134 (10)	0.0107 (9)	0.0120 (9)	0.0048 (9)	0.0000 (9)	0.0009 (9)
C7C	0.0146 (11)	0.0105 (10)	0.0116 (9)	0.0048 (9)	0.0001 (9)	0.0011 (9)
N8C	0.0143 (11)	0.0110 (10)	0.0141 (10)	0.0056 (9)	−0.0020 (9)	0.0008 (9)
O71C	0.0163 (17)	0.0111 (17)	0.0112 (15)	0.0028 (14)	−0.0004 (14)	0.0008 (14)
O72C	0.0175 (18)	0.0132 (16)	0.0120 (14)	0.0039 (14)	0.0018 (14)	0.0032 (13)
O81C	0.0155 (16)	0.0121 (13)	0.0169 (15)	0.0071 (13)	−0.0051 (14)	0.0013 (13)
O82C	0.0166 (17)	0.0137 (14)	0.0206 (16)	0.0035 (14)	−0.0049 (14)	0.0030 (13)
C1H	0.0137 (8)	0.0106 (7)	0.0120 (7)	0.0051 (7)	0.0002 (7)	0.0010 (6)
C2H	0.0139 (10)	0.0108 (9)	0.0130 (9)	0.0055 (8)	−0.0005 (8)	0.0009 (8)
C3H	0.0139 (11)	0.0109 (10)	0.0129 (10)	0.0055 (10)	−0.0002 (9)	0.0007 (9)
C4H	0.0138 (11)	0.0110 (11)	0.0129 (10)	0.0054 (10)	−0.0001 (10)	0.0005 (10)
C5H	0.0136 (11)	0.0109 (10)	0.0125 (10)	0.0052 (10)	0.0000 (9)	0.0007 (10)
C6H	0.0135 (10)	0.0107 (10)	0.0122 (10)	0.0050 (9)	0.0001 (9)	0.0008 (9)
C7H	0.0145 (11)	0.0107 (10)	0.0117 (10)	0.0048 (9)	0.0003 (9)	0.0012 (9)
N8H	0.0144 (12)	0.0112 (10)	0.0145 (11)	0.0054 (10)	−0.0015 (10)	0.0010 (10)
O71H	0.0151 (19)	0.0105 (19)	0.0114 (17)	0.0045 (18)	0.0002 (17)	0.0007 (17)
O72H	0.0153 (19)	0.0113 (18)	0.0118 (17)	0.0051 (18)	0.0017 (18)	0.0016 (17)
O81H	0.0151 (19)	0.0116 (18)	0.0159 (19)	0.0066 (18)	−0.0030 (18)	0.0004 (18)
O82H	0.0154 (18)	0.0126 (16)	0.0167 (17)	0.0047 (16)	−0.0033 (16)	0.0022 (16)
O1W	0.013 (2)	0.019 (2)	0.018 (2)	0.0011 (17)	−0.0015 (17)	0.0087 (16)
O2W	0.018 (2)	0.014 (2)	0.015 (2)	0.0025 (17)	0.0007 (17)	−0.0002 (15)
O3W	0.014 (2)	0.014 (2)	0.018 (2)	0.0018 (16)	−0.0006 (16)	−0.0010 (15)
O4W	0.011 (2)	0.017 (2)	0.0113 (18)	−0.0006 (16)	−0.0011 (15)	0.0043 (15)

### Geometric parameters (Å, º)

**Table:**

N1A—H1AA	0.9100	C4B—C5B	1.399 (8)
N1A—H1AB	0.9100	C5B—H5B	0.9500
N1A—Cu1A	1.991 (6)	C5B—C6B	1.379 (8)
N1A—C11A	1.487 (9)	C6B—H6B	0.9500
Cu1A—N2A	2.044 (5)	C7B—O71B	1.242 (8)
Cu1A—N3A	2.012 (6)	C7B—O72B	1.261 (8)
Cu1A—N4A	2.017 (5)	N8B—O81B	1.231 (8)
Cu1A—O1W	2.621 (5)	N8B—O82B	1.244 (8)
Cu1A—O4W	2.599 (5)	C1G—C2G	1.400 (5)
N2A—H2AA	0.9100	C1G—C6G	1.391 (8)
N2A—H2AB	0.9100	C1G—C7G	1.524 (9)
N2A—C12A	1.480 (9)	C2G—C3G	1.383 (8)
N3A—H3AA	0.9100	C2G—N8G	1.459 (9)
N3A—H3AB	0.9100	C3G—H3G	0.9500
N3A—C13A	1.479 (9)	C3G—C4G	1.393 (9)
N4A—H4AA	0.9100	C4G—H4G	0.9500
N4A—H4AB	0.9100	C4G—C5G	1.389 (10)
N4A—C14A	1.474 (9)	C5G—H5G	0.9500
C11A—H11A	0.9900	C5G—C6G	1.389 (9)
C11A—H11B	0.9900	C6G—H6G	0.9500
C11A—C12A	1.510 (9)	C7G—O71G	1.243 (9)
C12A—H12A	0.9900	C7G—O72G	1.263 (9)
C12A—H12B	0.9900	N8G—O81G	1.239 (9)
C13A—H13A	0.9900	N8G—O82G	1.235 (9)
C13A—H13B	0.9900	C1A—C2A	1.390 (8)
C13A—C14A	1.531 (9)	C1A—C6A	1.400 (8)
C14A—H14A	0.9900	C1A—C7A	1.517 (8)
C14A—H14B	0.9900	C2A—C3A	1.379 (9)
C1D—C2D	1.357 (10)	C2A—N8A	1.463 (9)
C1D—C6D	1.416 (10)	C3A—H3A	0.9500
C1D—C7D	1.492 (8)	C3A—C4A	1.382 (10)
C2D—C3D	1.407 (10)	C4A—H4A	0.9500
C2D—N8D	1.439 (11)	C4A—C5A	1.393 (10)
C3D—H3D	0.9500	C5A—H5A	0.9500
C3D—C4D	1.366 (14)	C5A—C6A	1.387 (9)
C4D—H4D	0.9500	C6A—H6A	0.9500
C4D—C5D	1.319 (14)	C7A—O71A	1.249 (8)
C5D—H5D	0.9500	C7A—O72A	1.271 (8)
C5D—C6D	1.402 (11)	N8A—O81A	1.239 (8)
C6D—H6D	0.9500	N8A—O82A	1.244 (8)
C7D—O71D	1.277 (10)	C1F—C2F	1.414 (9)
C7D—O72D	1.238 (9)	C1F—C6F	1.398 (9)
N8D—O81D	1.234 (9)	C1F—C7F	1.514 (9)
N8D—O82D	1.225 (9)	C2F—C3F	1.390 (10)
C1E—C2E	1.405 (11)	C2F—N8F	1.480 (10)
C1E—C6E	1.395 (11)	C3F—H3F	0.9500
C1E—C7E	1.492 (8)	C3F—C4F	1.390 (11)
C2E—C3E	1.414 (12)	C4F—H4F	0.9500
C2E—N8E	1.467 (12)	C4F—C5F	1.389 (12)
C3E—H3E	0.9500	C5F—H5F	0.9500
C3E—C4E	1.385 (15)	C5F—C6F	1.389 (10)
C4E—H4E	0.9500	C6F—H6F	0.9500
C4E—C5E	1.315 (16)	C7F—O71F	1.247 (9)
C5E—H5E	0.9500	C7F—O72F	1.268 (9)
C5E—C6E	1.395 (13)	N8F—O81F	1.231 (10)
C6E—H6E	0.9500	N8F—O82F	1.238 (10)
C7E—O71E	1.277 (11)	C1C—C2C	1.386 (8)
C7E—O72E	1.252 (11)	C1C—C6C	1.388 (7)
N8E—O81E	1.223 (11)	C1C—C7C	1.519 (8)
N8E—O82E	1.226 (11)	C2C—C3C	1.386 (9)
N1B—H1BA	0.9100	C2C—N8C	1.479 (8)
N1B—H1BB	0.9100	C3C—H3C	0.9500
N1B—Cu1B	1.999 (6)	C3C—C4C	1.377 (9)
N1B—C11B	1.478 (9)	C4C—H4C	0.9500
Cu1B—N2B	2.050 (5)	C4C—C5C	1.411 (10)
Cu1B—N3B	2.004 (6)	C5C—H5C	0.9500
Cu1B—N4B	2.031 (5)	C5C—C6C	1.382 (8)
Cu1B—O2W	2.557 (5)	C6C—H6C	0.9500
Cu1B—O3W	2.564 (5)	C7C—O71C	1.275 (8)
N2B—H2BA	0.9100	C7C—O72C	1.249 (8)
N2B—H2BB	0.9100	N8C—O81C	1.239 (8)
N2B—C12B	1.497 (9)	N8C—O82C	1.233 (8)
N3B—H3BA	0.9100	C1H—C2H	1.386 (13)
N3B—H3BB	0.9100	C1H—C6H	1.385 (13)
N3B—C13B	1.475 (9)	C1H—C7H	1.529 (13)
N4B—H4BA	0.9100	C2H—C3H	1.380 (14)
N4B—H4BB	0.9100	C2H—N8H	1.490 (14)
N4B—C14B	1.468 (10)	C3H—H3H	0.9500
C11B—H11C	0.9900	C3H—C4H	1.366 (16)
C11B—H11D	0.9900	C4H—H4H	0.9500
C11B—C12B	1.487 (10)	C4H—C5H	1.412 (15)
C12B—H12C	0.9900	C5H—H5H	0.9500
C12B—H12D	0.9900	C5H—C6H	1.393 (14)
C13B—H13C	0.9900	C6H—H6H	0.9500
C13B—H13D	0.9900	C7H—O71H	1.281 (14)
C13B—C14B	1.517 (11)	C7H—O72H	1.260 (14)
C14B—H14C	0.9900	N8H—O81H	1.251 (15)
C14B—H14D	0.9900	N8H—O82H	1.242 (14)
C1B—C2B	1.400 (5)	O1W—H1WA	0.8505
C1B—C6B	1.388 (7)	O1W—H1WB	0.8491
C1B—C7B	1.521 (8)	O2W—H2WA	0.8734
C2B—C3B	1.375 (7)	O2W—H2WB	1.0656
C2B—N8B	1.475 (8)	O3W—H3WA	0.8500
C3B—H3B	0.9500	O3W—H3WB	0.8478
C3B—C4B	1.387 (8)	O4W—H4WA	0.8497
C4B—H4B	0.9500	O4W—H4WB	0.8500
			
H1AA—N1A—H1AB	108.4	H13C—C13B—H13D	108.6
Cu1A—N1A—H1AA	110.0	C14B—C13B—H13C	110.3
Cu1A—N1A—H1AB	110.0	C14B—C13B—H13D	110.3
C11A—N1A—H1AA	110.0	N4B—C14B—C13B	109.1 (6)
C11A—N1A—H1AB	110.0	N4B—C14B—H14C	109.9
C11A—N1A—Cu1A	108.6 (4)	N4B—C14B—H14D	109.9
N1A—Cu1A—N2A	85.1 (2)	C13B—C14B—H14C	109.9
N1A—Cu1A—N3A	178.9 (2)	C13B—C14B—H14D	109.9
N1A—Cu1A—N4A	94.7 (2)	H14C—C14B—H14D	108.3
N1A—Cu1A—O1W	91.18 (19)	C2B—C1B—C7B	121.7 (11)
N1A—Cu1A—O4W	91.14 (19)	C6B—C1B—C2B	114.5 (5)
N2A—Cu1A—O1W	83.3 (2)	C6B—C1B—C7B	123.7 (11)
N2A—Cu1A—O4W	97.5 (2)	C1B—C2B—N8B	117.3 (5)
N3A—Cu1A—N2A	95.1 (2)	C3B—C2B—C1B	126.8 (6)
N3A—Cu1A—N4A	85.2 (2)	C3B—C2B—N8B	115.8 (6)
N3A—Cu1A—O1W	89.89 (19)	C2B—C3B—H3B	121.8
N3A—Cu1A—O4W	87.80 (19)	C2B—C3B—C4B	116.5 (7)
N4A—Cu1A—N2A	178.6 (3)	C4B—C3B—H3B	121.8
N4A—Cu1A—O1W	95.3 (2)	C3B—C4B—H4B	120.5
N4A—Cu1A—O4W	83.9 (2)	C3B—C4B—C5B	119.0 (8)
O4W—Cu1A—O1W	177.6 (2)	C5B—C4B—H4B	120.5
Cu1A—N2A—H2AA	110.2	C4B—C5B—H5B	118.9
Cu1A—N2A—H2AB	110.2	C6B—C5B—C4B	122.1 (8)
H2AA—N2A—H2AB	108.5	C6B—C5B—H5B	118.9
C12A—N2A—Cu1A	107.4 (4)	C1B—C6B—H6B	119.5
C12A—N2A—H2AA	110.2	C5B—C6B—C1B	120.9 (7)
C12A—N2A—H2AB	110.2	C5B—C6B—H6B	119.5
Cu1A—N3A—H3AA	110.0	O71B—C7B—C1B	118.3 (9)
Cu1A—N3A—H3AB	110.0	O71B—C7B—O72B	124.7 (9)
H3AA—N3A—H3AB	108.4	O72B—C7B—C1B	117.0 (9)
C13A—N3A—Cu1A	108.3 (4)	O81B—N8B—C2B	119.5 (8)
C13A—N3A—H3AA	110.0	O81B—N8B—O82B	121.7 (9)
C13A—N3A—H3AB	110.0	O82B—N8B—C2B	118.8 (7)
Cu1A—N4A—H4AA	110.0	C2G—C1G—C7G	120.1 (14)
Cu1A—N4A—H4AB	110.0	C6G—C1G—C2G	122.7 (6)
H4AA—N4A—H4AB	108.4	C6G—C1G—C7G	116.9 (15)
C14A—N4A—Cu1A	108.4 (4)	C1G—C2G—N8G	122.9 (6)
C14A—N4A—H4AA	110.0	C3G—C2G—C1G	115.8 (7)
C14A—N4A—H4AB	110.0	C3G—C2G—N8G	121.2 (7)
N1A—C11A—H11A	110.0	C2G—C3G—H3G	119.4
N1A—C11A—H11B	110.0	C2G—C3G—C4G	121.2 (10)
N1A—C11A—C12A	108.4 (6)	C4G—C3G—H3G	119.4
H11A—C11A—H11B	108.4	C3G—C4G—H4G	118.5
C12A—C11A—H11A	110.0	C5G—C4G—C3G	123.1 (11)
C12A—C11A—H11B	110.0	C5G—C4G—H4G	118.5
N2A—C12A—C11A	107.2 (5)	C4G—C5G—H5G	122.1
N2A—C12A—H12A	110.3	C4G—C5G—C6G	115.8 (11)
N2A—C12A—H12B	110.3	C6G—C5G—H5G	122.1
C11A—C12A—H12A	110.3	C1G—C6G—H6G	119.4
C11A—C12A—H12B	110.3	C5G—C6G—C1G	121.2 (10)
H12A—C12A—H12B	108.5	C5G—C6G—H6G	119.4
N3A—C13A—H13A	110.1	O71G—C7G—C1G	112.5 (12)
N3A—C13A—H13B	110.1	O71G—C7G—O72G	128.1 (12)
N3A—C13A—C14A	107.9 (6)	O72G—C7G—C1G	119.0 (13)
H13A—C13A—H13B	108.4	O81G—N8G—C2G	117.8 (11)
C14A—C13A—H13A	110.1	O82G—N8G—C2G	114.6 (10)
C14A—C13A—H13B	110.1	O82G—N8G—O81G	126.9 (12)
N4A—C14A—C13A	107.2 (5)	C2A—C1A—C6A	117.3 (6)
N4A—C14A—H14A	110.3	C2A—C1A—C7A	125.6 (7)
N4A—C14A—H14B	110.3	C6A—C1A—C7A	116.6 (8)
C13A—C14A—H14A	110.3	C1A—C2A—N8A	118.5 (6)
C13A—C14A—H14B	110.3	C3A—C2A—C1A	124.1 (7)
H14A—C14A—H14B	108.5	C3A—C2A—N8A	117.0 (7)
C2D—C1D—C6D	116.1 (7)	C2A—C3A—H3A	121.4
C2D—C1D—C7D	128.2 (6)	C2A—C3A—C4A	117.3 (8)
C6D—C1D—C7D	115.5 (6)	C4A—C3A—H3A	121.4
C1D—C2D—C3D	123.4 (8)	C3A—C4A—H4A	119.6
C1D—C2D—N8D	117.6 (7)	C3A—C4A—C5A	120.8 (8)
C3D—C2D—N8D	118.8 (8)	C5A—C4A—H4A	119.6
C2D—C3D—H3D	120.9	C4A—C5A—H5A	119.7
C4D—C3D—C2D	118.2 (10)	C6A—C5A—C4A	120.6 (8)
C4D—C3D—H3D	120.9	C6A—C5A—H5A	119.7
C3D—C4D—H4D	119.6	C1A—C6A—H6A	120.1
C5D—C4D—C3D	120.7 (9)	C5A—C6A—C1A	119.8 (8)
C5D—C4D—H4D	119.6	C5A—C6A—H6A	120.1
C4D—C5D—H5D	119.1	O71A—C7A—C1A	116.5 (8)
C4D—C5D—C6D	121.8 (10)	O71A—C7A—O72A	125.7 (9)
C6D—C5D—H5D	119.1	O72A—C7A—C1A	117.5 (9)
C1D—C6D—H6D	120.2	O81A—N8A—C2A	119.9 (8)
C5D—C6D—C1D	119.7 (9)	O81A—N8A—O82A	122.5 (8)
C5D—C6D—H6D	120.2	O82A—N8A—C2A	117.6 (7)
O71D—C7D—C1D	115.1 (10)	C2F—C1F—C7F	120.1 (10)
O72D—C7D—C1D	118.9 (8)	C6F—C1F—C2F	115.7 (7)
O72D—C7D—O71D	126.0 (11)	C6F—C1F—C7F	124.0 (11)
O81D—N8D—C2D	118.2 (9)	C1F—C2F—N8F	119.8 (8)
O82D—N8D—C2D	116.1 (8)	C3F—C2F—C1F	124.0 (9)
O82D—N8D—O81D	125.6 (9)	C3F—C2F—N8F	116.1 (9)
C2E—C1E—C7E	118.8 (7)	C2F—C3F—H3F	120.7
C6E—C1E—C2E	113.1 (8)	C2F—C3F—C4F	118.5 (11)
C6E—C1E—C7E	128.0 (8)	C4F—C3F—H3F	120.7
C1E—C2E—C3E	123.3 (10)	C3F—C4F—H4F	120.5
C1E—C2E—N8E	122.3 (9)	C5F—C4F—C3F	118.9 (11)
C3E—C2E—N8E	114.2 (9)	C5F—C4F—H4F	120.5
C2E—C3E—H3E	120.4	C4F—C5F—H5F	119.0
C4E—C3E—C2E	119.3 (12)	C4F—C5F—C6F	122.0 (11)
C4E—C3E—H3E	120.4	C6F—C5F—H5F	119.0
C3E—C4E—H4E	120.6	C1F—C6F—H6F	119.6
C5E—C4E—C3E	118.8 (11)	C5F—C6F—C1F	120.9 (11)
C5E—C4E—H4E	120.6	C5F—C6F—H6F	119.6
C4E—C5E—H5E	118.8	O71F—C7F—C1F	116.2 (11)
C4E—C5E—C6E	122.4 (12)	O71F—C7F—O72F	126.6 (14)
C6E—C5E—H5E	118.8	O72F—C7F—C1F	116.1 (12)
C1E—C6E—C5E	123.2 (11)	O81F—N8F—C2F	121.7 (11)
C1E—C6E—H6E	118.4	O81F—N8F—O82F	120.4 (11)
C5E—C6E—H6E	118.4	O82F—N8F—C2F	117.8 (11)
O71E—C7E—C1E	119.3 (12)	C2C—C1C—C6C	117.1 (5)
O72E—C7E—C1E	112.8 (10)	C2C—C1C—C7C	124.6 (7)
O72E—C7E—O71E	127.8 (15)	C6C—C1C—C7C	117.8 (7)
O81E—N8E—C2E	118.5 (11)	C1C—C2C—C3C	123.4 (6)
O81E—N8E—O82E	122.1 (12)	C1C—C2C—N8C	119.1 (5)
O82E—N8E—C2E	119.4 (11)	C3C—C2C—N8C	117.3 (5)
H1BA—N1B—H1BB	108.4	C2C—C3C—H3C	120.4
Cu1B—N1B—H1BA	110.1	C4C—C3C—C2C	119.1 (6)
Cu1B—N1B—H1BB	110.1	C4C—C3C—H3C	120.4
C11B—N1B—H1BA	110.1	C3C—C4C—H4C	120.7
C11B—N1B—H1BB	110.1	C3C—C4C—C5C	118.5 (6)
C11B—N1B—Cu1B	108.1 (4)	C5C—C4C—H4C	120.7
N1B—Cu1B—N2B	84.8 (2)	C4C—C5C—H5C	119.4
N1B—Cu1B—N3B	179.5 (3)	C6C—C5C—C4C	121.1 (6)
N1B—Cu1B—N4B	94.8 (2)	C6C—C5C—H5C	119.4
N1B—Cu1B—O2W	90.9 (2)	C1C—C6C—H6C	119.7
N1B—Cu1B—O3W	85.4 (2)	C5C—C6C—C1C	120.7 (6)
N2B—Cu1B—O2W	94.9 (2)	C5C—C6C—H6C	119.7
N2B—Cu1B—O3W	85.2 (2)	O71C—C7C—C1C	115.8 (7)
N3B—Cu1B—N2B	95.4 (2)	O72C—C7C—C1C	116.8 (7)
N3B—Cu1B—N4B	85.0 (2)	O72C—C7C—O71C	126.8 (7)
N3B—Cu1B—O2W	89.6 (2)	O81C—N8C—C2C	116.2 (6)
N3B—Cu1B—O3W	94.1 (2)	O82C—N8C—C2C	118.7 (6)
N4B—Cu1B—N2B	179.3 (3)	O82C—N8C—O81C	125.0 (6)
N4B—Cu1B—O2W	84.6 (2)	C2H—C1H—C7H	116 (2)
N4B—Cu1B—O3W	95.3 (2)	C6H—C1H—C2H	121.2 (12)
O2W—Cu1B—O3W	176.3 (2)	C6H—C1H—C7H	123 (2)
Cu1B—N2B—H2BA	110.3	C1H—C2H—N8H	121.7 (14)
Cu1B—N2B—H2BB	110.3	C3H—C2H—C1H	119.2 (14)
H2BA—N2B—H2BB	108.5	C3H—C2H—N8H	119.1 (14)
C12B—N2B—Cu1B	107.1 (4)	C2H—C3H—H3H	119.4
C12B—N2B—H2BA	110.3	C4H—C3H—C2H	121.2 (18)
C12B—N2B—H2BB	110.3	C4H—C3H—H3H	119.4
Cu1B—N3B—H3BA	109.9	C3H—C4H—H4H	120.5
Cu1B—N3B—H3BB	109.9	C3H—C4H—C5H	119.0 (18)
H3BA—N3B—H3BB	108.3	C5H—C4H—H4H	120.5
C13B—N3B—Cu1B	108.9 (4)	C4H—C5H—H5H	119.7
C13B—N3B—H3BA	109.9	C6H—C5H—C4H	120.6 (19)
C13B—N3B—H3BB	109.9	C6H—C5H—H5H	119.7
Cu1B—N4B—H4BA	110.2	C1H—C6H—C5H	118.3 (17)
Cu1B—N4B—H4BB	110.2	C1H—C6H—H6H	120.8
H4BA—N4B—H4BB	108.5	C5H—C6H—H6H	120.8
C14B—N4B—Cu1B	107.4 (4)	O71H—C7H—C1H	120 (2)
C14B—N4B—H4BA	110.2	O72H—C7H—C1H	117.4 (19)
C14B—N4B—H4BB	110.2	O72H—C7H—O71H	119 (3)
N1B—C11B—H11C	110.1	O81H—N8H—C2H	119.9 (17)
N1B—C11B—H11D	110.1	O82H—N8H—C2H	116.5 (17)
N1B—C11B—C12B	108.0 (6)	O82H—N8H—O81H	123.5 (18)
H11C—C11B—H11D	108.4	Cu1A—O1W—H1WA	124.2
C12B—C11B—H11C	110.1	Cu1A—O1W—H1WB	101.4
C12B—C11B—H11D	110.1	H1WA—O1W—H1WB	109.5
N2B—C12B—H12C	110.1	Cu1B—O2W—H2WA	82.9
N2B—C12B—H12D	110.1	Cu1B—O2W—H2WB	112.6
C11B—C12B—N2B	107.9 (5)	H2WA—O2W—H2WB	90.8
C11B—C12B—H12C	110.1	Cu1B—O3W—H3WA	125.0
C11B—C12B—H12D	110.1	Cu1B—O3W—H3WB	106.4
H12C—C12B—H12D	108.4	H3WA—O3W—H3WB	92.7
N3B—C13B—H13C	110.3	Cu1A—O4W—H4WA	99.0
N3B—C13B—H13D	110.3	Cu1A—O4W—H4WB	109.8
N3B—C13B—C14B	106.9 (6)	H4WA—O4W—H4WB	109.5
			
N1A—C11A—C12A—N2A	53.3 (7)	C6G—C1G—C7G—O71G	118 (3)
Cu1A—N1A—C11A—C12A	−39.2 (6)	C6G—C1G—C7G—O72G	−56 (4)
Cu1A—N2A—C12A—C11A	−40.7 (6)	C7G—C1G—C2G—C3G	167.7 (17)
Cu1A—N3A—C13A—C14A	39.2 (6)	C7G—C1G—C2G—N8G	−14.9 (17)
Cu1A—N4A—C14A—C13A	40.1 (6)	C7G—C1G—C6G—C5G	−170.9 (19)
N3A—C13A—C14A—N4A	−52.8 (7)	N8G—C2G—C3G—C4G	−173.8 (14)
C1D—C2D—C3D—C4D	−2 (2)	C1A—C2A—C3A—C4A	1.8 (19)
C1D—C2D—N8D—O81D	31.0 (18)	C1A—C2A—N8A—O81A	150.2 (11)
C1D—C2D—N8D—O82D	−153.0 (12)	C1A—C2A—N8A—O82A	−28.7 (16)
C2D—C1D—C6D—C5D	−2.9 (17)	C2A—C1A—C6A—C5A	1.4 (16)
C2D—C1D—C7D—O71D	−130.2 (19)	C2A—C1A—C7A—O71A	−44 (2)
C2D—C1D—C7D—O72D	47.0 (15)	C2A—C1A—C7A—O72A	130.7 (14)
C2D—C3D—C4D—C5D	−1.6 (19)	C2A—C3A—C4A—C5A	0.4 (16)
C3D—C2D—N8D—O81D	−143.0 (13)	C3A—C2A—N8A—O81A	−36.2 (17)
C3D—C2D—N8D—O82D	33.1 (18)	C3A—C2A—N8A—O82A	144.9 (11)
C3D—C4D—C5D—C6D	2.4 (19)	C3A—C4A—C5A—C6A	−1.5 (16)
C4D—C5D—C6D—C1D	0.0 (19)	C4A—C5A—C6A—C1A	0.6 (17)
C6D—C1D—C2D—C3D	4 (2)	C6A—C1A—C2A—C3A	−2.7 (18)
C6D—C1D—C2D—N8D	−170.0 (12)	C6A—C1A—C2A—N8A	170.4 (10)
C6D—C1D—C7D—O71D	55.8 (19)	C6A—C1A—C7A—O71A	127.6 (14)
C6D—C1D—C7D—O72D	−127.0 (11)	C6A—C1A—C7A—O72A	−57.6 (18)
C7D—C1D—C2D—C3D	−170.2 (11)	C7A—C1A—C2A—C3A	168.9 (12)
C7D—C1D—C2D—N8D	16.1 (19)	C7A—C1A—C2A—N8A	−17.9 (18)
C7D—C1D—C6D—C5D	171.8 (10)	C7A—C1A—C6A—C5A	−171.0 (11)
N8D—C2D—C3D—C4D	172.0 (12)	N8A—C2A—C3A—C4A	−171.4 (10)
C1E—C2E—C3E—C4E	−2 (3)	C1F—C2F—C3F—C4F	1 (3)
C1E—C2E—N8E—O81E	29 (3)	C1F—C2F—N8F—O81F	146.7 (16)
C1E—C2E—N8E—O82E	−149.5 (17)	C1F—C2F—N8F—O82F	−29 (2)
C2E—C1E—C6E—C5E	−1 (2)	C2F—C1F—C6F—C5F	3 (2)
C2E—C1E—C7E—O71E	−127 (3)	C2F—C1F—C7F—O71F	−58 (3)
C2E—C1E—C7E—O72E	57.0 (16)	C2F—C1F—C7F—O72F	133 (2)
C2E—C3E—C4E—C5E	1 (2)	C2F—C3F—C4F—C5F	−1 (3)
C3E—C2E—N8E—O81E	−145.5 (16)	C3F—C2F—N8F—O81F	−38 (2)
C3E—C2E—N8E—O82E	36 (2)	C3F—C2F—N8F—O82F	145.7 (17)
C3E—C4E—C5E—C6E	−1 (2)	C3F—C4F—C5F—C6F	2 (3)
C4E—C5E—C6E—C1E	1 (3)	C4F—C5F—C6F—C1F	−3 (3)
C6E—C1E—C2E—C3E	1 (2)	C6F—C1F—C2F—C3F	−2 (3)
C6E—C1E—C2E—N8E	−172.6 (17)	C6F—C1F—C2F—N8F	172.5 (16)
C6E—C1E—C7E—O71E	58 (3)	C6F—C1F—C7F—O71F	116 (2)
C6E—C1E—C7E—O72E	−118.5 (15)	C6F—C1F—C7F—O72F	−52 (3)
C7E—C1E—C2E—C3E	−174.9 (15)	C7F—C1F—C2F—C3F	172.9 (18)
C7E—C1E—C2E—N8E	11 (2)	C7F—C1F—C2F—N8F	−13 (2)
C7E—C1E—C6E—C5E	174.8 (12)	C7F—C1F—C6F—C5F	−172.0 (16)
N8E—C2E—C3E—C4E	172.7 (15)	N8F—C2F—C3F—C4F	−173.4 (16)
N1B—C11B—C12B—N2B	−54.4 (7)	C1C—C2C—C3C—C4C	−0.1 (12)
Cu1B—N1B—C11B—C12B	43.4 (6)	C1C—C2C—N8C—O81C	36.2 (10)
Cu1B—N2B—C12B—C11B	38.0 (6)	C1C—C2C—N8C—O82C	−147.5 (7)
Cu1B—N3B—C13B—C14B	−39.8 (6)	C2C—C1C—C6C—C5C	−0.5 (13)
Cu1B—N4B—C14B—C13B	−39.0 (7)	C2C—C1C—C7C—O71C	−140.6 (11)
N3B—C13B—C14B—N4B	52.7 (7)	C2C—C1C—C7C—O72C	48 (2)
C1B—C2B—C3B—C4B	−1.7 (14)	C2C—C3C—C4C—C5C	−1.2 (11)
C1B—C2B—N8B—O81B	−36.9 (11)	C3C—C2C—N8C—O81C	−139.0 (7)
C1B—C2B—N8B—O82B	145.1 (8)	C3C—C2C—N8C—O82C	37.3 (11)
C2B—C1B—C6B—C5B	2.6 (16)	C3C—C4C—C5C—C6C	1.7 (11)
C2B—C1B—C7B—O71B	−60 (3)	C4C—C5C—C6C—C1C	−0.8 (14)
C2B—C1B—C7B—O72B	120 (2)	C6C—C1C—C2C—C3C	1.0 (13)
C2B—C3B—C4B—C5B	3.2 (15)	C6C—C1C—C2C—N8C	−173.9 (8)
C3B—C2B—N8B—O81B	140.4 (10)	C6C—C1C—C7C—O71C	47.3 (18)
C3B—C2B—N8B—O82B	−37.5 (12)	C6C—C1C—C7C—O72C	−124.6 (13)
C3B—C4B—C5B—C6B	−1.9 (16)	C7C—C1C—C2C—C3C	−171.2 (10)
C4B—C5B—C6B—C1B	−1.2 (19)	C7C—C1C—C2C—N8C	13.9 (14)
C6B—C1B—C2B—C3B	−1.2 (12)	C7C—C1C—C6C—C5C	172.3 (10)
C6B—C1B—C2B—N8B	175.8 (9)	N8C—C2C—C3C—C4C	174.8 (7)
C6B—C1B—C7B—O71B	116 (2)	C1H—C2H—C3H—C4H	−9 (5)
C6B—C1B—C7B—O72B	−63 (3)	C1H—C2H—N8H—O81H	32 (4)
C7B—C1B—C2B—C3B	175.6 (12)	C1H—C2H—N8H—O82H	−150 (3)
C7B—C1B—C2B—N8B	−7.5 (13)	C2H—C1H—C6H—C5H	−5 (5)
C7B—C1B—C6B—C5B	−174.1 (13)	C2H—C1H—C7H—O71H	−120 (5)
N8B—C2B—C3B—C4B	−178.7 (9)	C2H—C1H—C7H—O72H	39 (6)
C1G—C2G—C3G—C4G	3.7 (19)	C2H—C3H—C4H—C5H	5 (5)
C1G—C2G—N8G—O81G	−32.4 (17)	C3H—C2H—N8H—O81H	−145 (3)
C1G—C2G—N8G—O82G	156.9 (11)	C3H—C2H—N8H—O82H	32 (4)
C2G—C1G—C6G—C5G	2 (3)	C3H—C4H—C5H—C6H	−1 (5)
C2G—C1G—C7G—O71G	−56 (4)	C4H—C5H—C6H—C1H	1 (5)
C2G—C1G—C7G—O72G	131 (3)	C6H—C1H—C2H—C3H	9 (4)
C2G—C3G—C4G—C5G	1 (3)	C6H—C1H—C2H—N8H	−169 (3)
C3G—C2G—N8G—O81G	144.9 (14)	C6H—C1H—C7H—O71H	56 (7)
C3G—C2G—N8G—O82G	−25.7 (18)	C6H—C1H—C7H—O72H	−145 (5)
C3G—C4G—C5G—C6G	−4 (2)	C7H—C1H—C2H—C3H	−175 (3)
C4G—C5G—C6G—C1G	2 (3)	C7H—C1H—C2H—N8H	7 (4)
C6G—C1G—C2G—C3G	−5.5 (16)	C7H—C1H—C6H—C5H	179 (4)
C6G—C1G—C2G—N8G	172.0 (14)	N8H—C2H—C3H—C4H	169 (3)

### Hydrogen-bond geometry (Å, º)

**Table:**

*D*—H···*A*	*D*—H	H···*A*	*D*···*A*	*D*—H···*A*
N1*A*—H1*AA*···O71*B*	0.91	1.98	2.881 (14)	172
N3*A*—H3*AB*···O72*D*	0.91	1.90	2.809 (11)	174
N1*B*—H1*BB*···O72*C*	0.91	1.99	2.902 (8)	174
N1*B*—H1*BB*···O72*H*	0.91	1.86	2.77 (2)	172
N2*B*—H2*BB*···O72*A*	0.91	2.02	2.874 (12)	155
N3*B*—H3*BA*···O71*A*	0.91	1.89	2.795 (10)	176
N4*B*—H4*BA*···O71*H*	0.91	1.93	2.79 (3)	158
O1*W*—H1*WA*···O72*A*	0.85	1.97	2.794 (13)	164
O1*W*—H1*WA*···O72*F*	0.85	1.92	2.719 (18)	156
O1*W*—H1*WB*···O71*D*	0.85	1.94	2.763 (19)	162
O1*W*—H1*WB*···O71*E*	0.85	1.87	2.69 (3)	162
O2*W*—H2*WA*···O71*C*	0.87	1.98	2.739 (9)	145
O2*W*—H2*WA*···O71*H*	0.87	2.03	2.78 (3)	143
O2*W*—H2*WB*···O71*C*^i^	1.07	1.78	2.803 (10)	160
O2*W*—H2*WB*···O71*H*^i^	1.07	1.78	2.76 (3)	152
O3*W*—H3*WA*···O71*D*^ii^	0.85	1.92	2.739 (15)	160
O3*W*—H3*WA*···O71*E*^ii^	0.85	1.99	2.808 (19)	161
O3*W*—H3*WB*···O72*A*	0.85	2.04	2.750 (13)	141
O3*W*—H3*WB*···O72*F*	0.85	2.04	2.774 (19)	145
O4*W*—H4*WA*···O72*B*	0.85	1.91	2.753 (12)	173
O4*W*—H4*WA*···O72*G*	0.85	1.82	2.662 (17)	172
O4*W*—H4*WB*···O72*B*^iii^	0.85	1.90	2.726 (13)	164
O4*W*—H4*WB*···O72*G*^iii^	0.85	1.99	2.829 (17)	168

Symmetry codes: (i) *x*, −*y*, *z*−1/2; (ii) *x*, *y*, *z*+1; (iii) *x*, −*y*+1, *z*−1/2.
